# Is Annual Monitoring of Prolactin for Patients on Long Term Antipsychotics Being Completed and Results Acted Upon in Rochdale Community Mental Health Team?

**DOI:** 10.1192/bjo.2024.560

**Published:** 2024-08-01

**Authors:** Rania El-Nemr, Emily Melling, Saba Yasin

**Affiliations:** Pennine Care NHS Foundation Trust, Rochdale, United Kingdom

## Abstract

**Aims:**

National guidelines (NICE) recommend that prolactin should be monitored every 12 months for patients on antipsychotics, excluding patients on aripiprazole, clozapine, quetiapine or on doses of less than 20mg daily of olanzapine. The purpose of this audit was to investigate whether patients under our services who are prescribed antipsychotics implicated in causing hyperprolactinemia, were having regular annual prolactin measurements as per the guidelines and whether abnormal results were being actioned appropriately.

**Methods:**

A total of 61 patients were surveyed, as a random selection from the Outpatient Consultant case load in Rochdale CMHT. This was a retrospective analysis looking at annual prolactin measurements over 5 years between 01/01/2017 and 31/12/2022. This included all patients who had been stabilised on an antipsychotic for more than 2 years, and excluded patients on antipsychotics that did not cause significant prolactin rise (and so do not require annual prolactin measurements as per NICE guidelines).

**Results:**

Our results showed that the majority of patients were not having regular annual prolactin measurements, with only 3.3% of patients having prolactin measured annually 100% of the time. 23% of patients had no prolactin measurements at all while on antipsychotic treatment during the time period assessed. In cases were there was an elevated prolactin reading, only 15% of these readings had a documented action plan.

**Conclusion:**

This audit has demonstrated that the overall compliance with the NICE standards for annual prolactin monitoring for people on antipsychotic medication is of a poor standard, and we highlight possible reasons why this may not be done and areas for improvement.

